# Impact of nonpharmaceutical strategies on trends of COVID-19 in São Paulo State

**DOI:** 10.11606/s1518-8787.2021055003599

**Published:** 2021-07-27

**Authors:** Cristiane Ravagnani Fortaleza, Thomas Nogueira Vilches, Gabriel Berg de Almeida, Claudia Pio Ferreira, Lenice do Rosário de Souza, Carlos Magno Castelo Branco Fortaleza

**Affiliations:** I Universidade Estadual Paulista Faculdade de Medicina de Botucatu Departamento de Infectologia BotucatuSP Brasil Universidade Estadual Paulista. Faculdade de Medicina de Botucatu. Departamento de Infectologia. Botucatu, SP, Brasil; II Universidade Estadual Paulista Departamento de Bioestatística Instituto de Biociências da Botucatu BotucatuSP Brasil Universidade Estadual Paulista. Departamento de Bioestatística. Instituto de Biociências da Botucatu. Botucatu, SP, Brasil

**Keywords:** Coronavirus Infections, prevention & control, Facial Masks, Social Isolation, Local Strategies, Disease Transmission, Infectious

## Abstract

Interrupted time series analyses were conducted to measure the impact of social distancing policies (instituted on March 22, 2020) and of subsequent mandatory masking in the community (instituted on May 4, 2020) on the incidence and effective reproductive number of COVID-19 in São Paulo State, Brazil. Overall, the impact of social distancing both on incidence and Rt was greater than the incremental effect of mandatory masking. Those findings may reflect either a small impact of face masking or the loosening of social distancing after mandatory use of masks.

## INTRODUCTION

São Paulo state reported the first case of COVID-19 in Brazil, on February 25, 2020. As of February 2021, laboratory-confirmed cases totaled 9 million, with almost 240,000 deaths. Circa 20% of cases and deaths occurred in São Paulo, Brazil’s most populous state (45 million inhabitants). Geographic modeling^1^ and ecological analyzes^2^documented patterns of disease spread from the metropolitan area, where 39 cities harbor 21 million inhabitants, to the remaining 606 municipalities located in the inner areas of the state.

To contain the spread of SARS-Cov-2, the government of São Paulo decreed the closure of non-essential services and the restriction of public transport since March 22, 2020. Even though a study (presently available as PrePrint manuscript) suggests the effectiveness of that measure in preventing deaths and intensive care units (ICU) admissions^3^, data on its impact in the inner areas of the state is still lacking. Furthermore, a second decree from May 4, 2020 made the use of face masks in community settings mandatory.

Our study aimed at analyzing the impact of both control measures cited above on daily incidence rates (per 100,000 inhabitants) and daily estimates of the effective reproductive number (R_t_) of laboratory-confirmed COVID-19 cases in the metropolitan and inner areas of São Paulo State.

## METHODS

An ecological study based on time series analysis was conducted. Daily incidences between March 1 and July 4 were obtained from São Paulo State Epidemiological Surveillance Center open access database^a^. The daily incidence was based on the dates of case reporting, since information on the date of first symptoms was not readily available in the database. In order to calculate incidence rates, we used population data for the year 2020 recovered from the São Paulo State Foundation for Data Analysis^b^. The time series of cases was used to estimate R_t_, as described by Wallinga and Lipsitch^4^.

Interrupted time series (ITS) analysis was conducted using STATA 14 (Statacorp, College Station, TX), with segmented regression models that included both successive interventions (social distancing (#1) and universal masking (#2)). Those models fitted linear trends for each period (pre-intervention #1, between interventions, and post-intervention #2). In order to fulfill assumptions for the segmented regression analysis, we fitted linear trends for all periods (pre- and post-interventions). As usual for ITS analysis, our multivariable models assessed the adjusted impact of time trend, the intervention, and the interaction of those two variables (which measures the intervention long-term impact on time trends).

This study was approved by the Committee for Ethics in Research from Faculdade de Medicina de Botucatu (CAAE 32741320.0.0000.5411).

## RESULTS

### Impact of Social Distancing Normative

The Figure shows the results of ITS analysis. Social distancing had no immediate beneficial impact on COVID-19 incidence in the metropolitan area (linear regression coefficient [LRC] = -0.19; 95% confidence interval [95%CI] -0.15 to -0.53). On the other hand, it presented beneficial impact in the inner state (LRC = -0.41; 95%CI: -0.69 to -0.13)). Significant (p *<* 0.05) long term trend change was observed in both areas (metropolitan area, LCR = -0.08; 95%CI: -0.10 to -0.05; inner state, LRC, -0,06; 95%CI: -0.05 to -0.02). On the other hand, the immediate impact on R_t_ was significant for both metropolitan (LRC, -1.50; 95% CI, -1.88 to -1.11) and inner areas (LRC, -1.17; 95%CI: -1.57 to -0.77). A similar result was detected for long-term trend impact (metropolitan area, LRC, -0.08; 95%CI: -0.10 to -0.05; inner state, LRC, -0.002; 95%CI: -0.004 to -0.001).

**Figure:**
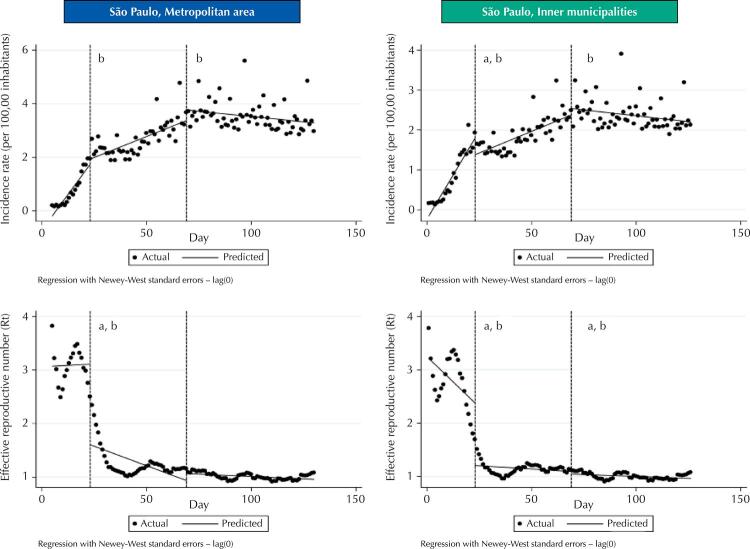
Graphical presentation of the impact of governmental interventions for social distancing and mandatory universal masking on trends of incidence and effective reproduction number (Rt) of laboratory-confirmed COVID-19 cases in the metropolitan area and inner municipalities of São Paulo State, Brazil.

Note. The two dashed vertical lines in the graphs represent, respectively, government decrees of social restriction (March 22) and universal use of masks in the community (May 4). Embedded in the graphics, regression coefficients for immediate impact and impact on time trends are presented. Beneficial impact of interventions with p-value < 0.05 are indicated in graphics: letter “a” for immediate impact and “b” for impact on long-term trend.

### Normative Mask Use

The incremental beneficial impact of universal masking was not immediate on overall incidence (metropolitan area, LRC, 0.40; 95%CI: 0.01 to 0.79; inner state, LRC, 0.16; 95%CI: -0.11 to 0.43), but we observed a long-term significant impact (P < 0.05) for both metropolitan area (LRC, -0.04; 95%CI: -0.05 to -0.02) and the inner state (LCR = -0.03; 95%CI: -0.04 to -0.02).

When the R_t_ values were studied, the metropolitan area presented neither immediate impact (LRC, 0.13; 95%CI: -0.01 to 0.27) nor a long-term benefit (LRC, 0.13; 95%CI: 0.01 to 0.20). However, for the inner state municipalities, the beneficial impact was both immediate (LRC, -1.17; 95%CI: -1.57 to -0.77) and on long-term trend (LRC, -0.002; 95%CI: -0.004 to -0.001).

### Interpretation of Findings

Data presented above demonstrate that, even though the immediate impact of normative for nonpharmaceutical measures varied, both policies were followed by a significant downward change in time trends of incidence and reproductive number (R_t_). The interpretation of R_t_ linear models suggests that the impact of the governmental decree of mandatory social distancing was greater than of the latter decree on universal masking.

## DISCUSSION

São Paulo state did not experience a lockdown in the strict sense of the word. Instead, the March 22 decree attempted to induce social distancing by closing non-essential services (e.g., stores) and restricting public transport (subway, metropolitan railways, and buses). ITS analysis demonstrates a variable but generally beneficial impact of that measure. This agrees with previous reports and modeling predictions^5^. The incremental benefit of mandatory universal masking was subtle, with an impact on incidence rates but not on daily R_t_ series.

However, our study has limitations. First, our incidence data was based on the day of notification, not that of the first symptoms. While this is not the ideal approach, imprecisions were diluted in long-term analysis, thus not threatening the study’s overall accuracy. Presumably voluntary individual protective behavior (including staying at home and using homemade masks) may have started before governmental decrees were issued. However, both regulations were extensively publicized in the press, television, and online media. Both shops and stores that did not close and persons not using masks in public places were subjected to heavy fines. Therefore, it seems plausible that social distancing and mask use increased after the governmental decrees. In an opposite direction, a gradual loosening of restrictive policies (following the so-called São Paulo Plan^c^) might have counterbalanced the impact of face masking. Briefly, those measures, initiated in June 2020 were slowly and heterogeneously applied. The State was divided into 22 administrative areas, which could open 0 to 40% of non-essential services, depending on standards of COVID-19 incidence and mortality, availability, and occupation of Intensive Care Unit beds. Municipalities were also allowed to implement additional restrictive measures, as aspect which increased the heterogeneity of the preventive policies. Recommendations changed every two weeks, depending on novel epidemiological data. To assess that slowly applied, geographically heterogeneous, and time-varying strategy was beyond the scope of this study. Also, the continuous rise in population mobility (as detected from the movement of mobile phones and reported in the São Paulo Government homepage^d^) was not included in our models, since we had access only to incomplete data. Still, this gradual rise probably increased the opportunity for transmission, underestimating the impact of the face mask decree. It is worth noting that, even though the effectiveness of nonpharmaceutical methods for preventing COVID-19 spread has been supported by systematic review^5^, the debate on their importance is far from solved. For an interesting approach to this issue, we suggest readers to consult the *British Medical Journal* blog^e^. Noticeably, this debate is wide enough to include issues like individual rights, environmental impacts (of discarded masks), and population compliance.

The increasing availability of laboratory testing may have influenced our findings. However, that increase was greater during the first months of pandemics in Brazil, so they were expected to underestimate the impact of the first intervention. Even though those aspects might influence the impact of nonpharmaceutical measures, they are real-life phenomena inherent to studies of effectiveness (as opposed to efficacy). Another limitation of our study was its ecological approach. Obviously, we did not assess the impact of staying at home or using cloth masks on individual risks of being infected by SARS-Cov-2. Our main objective was to study the collective impact of governmental policies.

A systematic review of nonpharmaceutical measures to prevent COVID-19, based solely on observational studies, concluded that social distancing, use of masks, and eye protection were beneficial^5^. Unfortunately, those results are limited by the heterogeneity of settings (healthcare and community) and of interventions. The analysis of benefits from the use of masks is particularly challenging. Previous studies point out to an expected greater efficacy of N95 respirators and surgical masks than that of cloth and/or homemade masks. It is worth noting that a recent systematic review with meta-analysis or randomized clinical trials found general protective impact of face masks^5^. However, protection was greater in healthcare settings than in the community. Also, the included studies assessed influenza and other respiratory viruses, and most of them tested surgical masks. Therefore, inferences have been largely based on analogy, and the benefits of cloth masks are far from straightforward.

## CONCLUSION

We found that governmental strategies based on nonpharmaceutical intervention were followed by slowing the evolution of pandemics in São Paulo State, Brazil. The effectiveness was greater for the first intervention (social distancing), with some incremental impact of mandatory use of face masks. Those findings may reflect either a small impact of face masking or the loosening of social distancing after mandatory use of masks. Either way, they contribute for directing public policies against COVID-19 in Brazil and other countries, in a period when the world is still far from achieving control of the current pandemics.

## Footnotes

a Secretaria de Estado da Saúde de São Paulo. Centro de Vigilância Epidemiológica “Prof Alexandre Vranjac”. São Paulo: CVE; s.d. Available from: www.cve.saude.sp.gov.br

b Fundação Sistema Estadual de Análise de Dados. São Paulo: SEADE; s.d. Available from: https://www.seade.gov.br/

c São Paulo (Estado). Plano São Paulo. São Paulo; sd. Available from: https://www.saopaulo.sp.gov.br/planosp/

d Centro de Contingência do Estado de São Paulo. SP contra o Novo Coronavirus. São Paulo; s.d. Available from: https://www.saopaulo.sp.gov.br/coronavirus/

e Margaret McCartney: We need better evidence on non-drug interventions for covid-19. The BMJ Opinion. 2020 Aug 28. Available from: https://blogs.bmj.com/bmj/2020/08/28/margaret-mccartney-we-need-better-evidence-on-non-drug-interventions-for-covid-19/
